# Use of an unmanned aerial vehicle for monitoring and prediction of oilseed rape crop performance

**DOI:** 10.1371/journal.pone.0294184

**Published:** 2023-11-10

**Authors:** Shara Ahmed, Catherine E. Nicholson, Simon R. Rutter, John R. Marshall, Justin J. Perry, John R. Dean

**Affiliations:** Department of Applied Sciences, Northumbria University, Ellison Building, Newcastle upon Tyne, United Kingdom; University of the Pacific, UNITED STATES

## Abstract

The flowering stage of oilseed rape (*Brassica napus* L.) is of vital interest in precision agriculture. It has been shown that data describing the flower production of oilseed rape (OSR), at stage 3, in spring can be used to predict seed yield at harvest. Traditional field-based techniques for assessing OSR flowers are based on a visual assessment which is subjective and time consuming. However, a high throughput phenotyping technique, using an unmanned aerial vehicle (UAV) with multispectral image (MSI) camera, was used to investigate the growth stages of OSR (in terms of crop height) and to quantify its flower production. A simplified approach using a normalised difference yellowness index (NDYI) was coupled with an iso-cluster classification method to quantify the number of OSR flower pixels and incorporate the data into an OSR seed yield estimation. The estimated OSR seed yield showed strong correlation with the actual OSR seed yield (R^2^ = 0.86), as determined using *in-situ* sensors mounted on the combine harvester. Also, using our approach allowed the variation in crop height to be assessed across all growing stages; the maximum crop height of 1.35 m OSR was observed at the flowering stage. This methodology is proposed for effectively predicting seed yield 3 months prior to harvesting.

## Introduction

Oilseed rape (OSR) (*Brassica napus* L.) is a widely cultivated crop worldwide due to its oil-rich seeds [1]. After harvesting, the seeds are crushed to liberate the highly desirable oil which has been consumed at 28–29 million tonnes per year worldwide for the past seven years [2]. In addition, the by-products are used for animal feed, biofuel, and medicine [3]. The UK’s Agriculture and Horticulture Development Board (AHDB) planting survey anticipated an OSR production of 1.04 Mt [4]. This decreased OSR production was identified to be due to extreme weather conditions, linked to climate change, and the cost of effective crop management [5]. To reverse this trend, a more cost-effective and forward-looking crop management system is needed. This research explores the use of an unmanned aerial vehicle (UAV) with multispectral image (MSI) camera precision for data collection, to assess the crop phenological growth stages of OSR. Ultimately, the goal is to provide data for OSR intervention strategies to maximise yield by timely and cost-effective application of fertilizers and pesticides.

**Fig 1:**
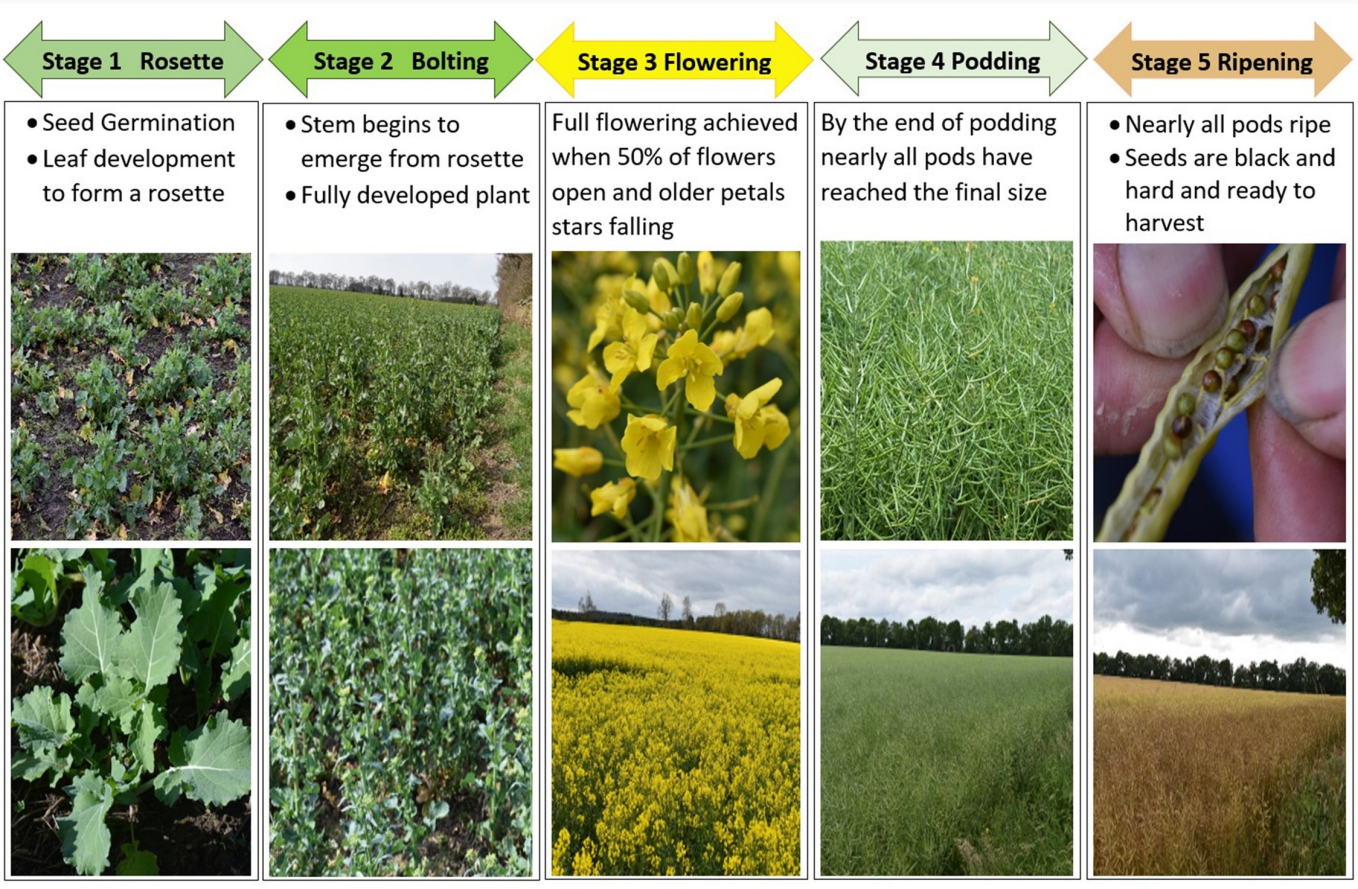
The phenological growth stages of oilseed rape.

The phenological growth stages of OSR have been codified, Fig 1 [6]. Stages 1 and 2 incorporate both seed germination and plant development. Flowering of the OSR occurs in stage 3, while in stages 4 and 5 OSR pod development and ripening take place. An important agronomic trait used through every phenological growth stage to assess development is the OSR plant height. The traditional, field-based method for estimating general plant height involves measuring the height of two or three plants from a single plot using a ruler [7]. This value is then used not only to monitor OSR plant development but also to inform decision making regarding the application of fertilizers to maximize the OSR oil yield [8]. Monitoring the flowering stage of OSR through to the maturation of the OSR pod allows a more accurate estimation of the seed yield [9, 10]. Traditionally, the collection and use of OSR flower, plant and pod data from field investigation is subjective, labour intensive and potentially destructive to crops [8]. Hence, the deployment of remote sensing techniques has become an area of interest in acquiring objective and accurate crop information.

The two main remote sensing platforms used to collect crop related data are satellites and unmanned aerial vehicles (UAVs) [10] which offer low spatial and high spectral resolution, respectively. Spatial resolution is the number of pixels incorporated to comprise an image by the ground sampling distance (GSD) of a sensor/camera. High spectral resolution is determined by the number of wavelength bands in the visible, near-infrared (NIR) and shortwave infrared which are collected. Recent studies have evaluated remote sensing techniques to estimate OSR crop height using a UAV integrated with a RGB and light detection and ranging (LiDAR) sensor [7, 8]. However, in these studies, data was gathered over a few days, limiting their flexibility and hence utility. Furthermore, a LiDAR data set can be extremely large and challenging to automatically interpret.

Specifically, the use of remote sensing approaches to predict OSR seed yield has been a growing area of interest over the last few years [11]. The distinctive visibility of the OSR flowers (Stage 3) can be distinguished using characteristic spectral bands by remote sensing platforms. Recent studies using satellite remote sensing platforms have proposed spectral indices which have been used to estimate the OSR seed yield [12–14]. However, low spatial resolution (e.g., 500 m/pixel) obtainable using satellite images diminishes the accuracy for smaller agricultural sites (e.g. 5–10 ha). Also, the hyperspectral satellite image data requires extensive training and automation to be readily implementable and hence adopted by end users, such as farmers or agronomists. Additionally, satellite platforms are significantly influenced by adverse weather conditions and cloud cover, which when coupled with low temporal resolution, can restrict data collection due to their inability to collect data within the same day. Due to these limitations, UAV remote sensing platforms have become the dominant option to estimate the OSR seed yield. Also, UAVs utilize consumer grade digital RGB cameras or multispectral cameras which simplify the data analysis process, are less susceptible to cloud cover and offer flexible temporal resolution where data can be collected multiple times in a day [15]

An estimate of the biomass of winter OSR using vegetation indices coupled with a random forest regression model based on gathered UAV multispectral images has been reported [16]. This study evaluated the potential of UAV to retrieve plot level information about a specific OSR crop profile. Further, UAV multispectral data has also shown promising results by predicting OSR seed yield from correlation with the leaf area index (LAI) during different phenological growth stages [17, 18]. However, these studies require time consuming field crop samples to be analysed in the laboratory for validation.

The OSR flowers provide spectral features which can be interpreted by the sensors used in remote sensing platforms. The yellowness of OSR flowers is potentially due to the carotenoid absorption of blue light and the reflectance of green and red spectral bands [9, 19]. Sulik and Long, 2015 reported that the ratio of green and blue spectral bands were strongly correlated (R^2^ = 0.87) to determine the number of OSR flowers [19]. They then proposed that a new normalised difference yellowness index (NDYI) based on the green and blue spectral bands was a significant OSR seed yield predictor [20]. Other studies which have combined multiple vegetation indices and image classification methods (e.g. k-means clustering and pixel level mixture analysis) have improved the identification of OSR flowers and hence improved seed yield prediction [10, 21]. Similarly, a study used image-based phenotyping using thresholding to classify winter (R^2^ = 0.84) and spring OSR flowers (R^2^ = 0.72) [22]. Additionally, a simplified approach using multi-series data, generated by NDYI-based flowering pixel, and classified by thresholding successfully estimated OSR seed yield [11]. However, the most relevant of these studies [11, 21], estimated OSR seed yield based on a single data set on a relatively small field area. In addition, the use of thresholding for classification can be highly inconsistent, and error prone, as each threshold value must be determined manually, and the unsupervised k-means image processing studies were not performed on NDYI derived maps [10, 21]. This has limited the understanding of the performance of combining an unsupervised classification model with NDYI to estimate OSR seed yield.

This study determines the OSR flower coverage on a typical field size, by NDYI, coupled with the simpler and precise image classification method of iso-clustering (using commercially available software). Iso-clustering classification is an automated unsupervised classification model that aims to segment similar clusters to groups by assigning distinctive pixel values. The iso-cluster classification uses the same principal as k-means clustering but is more automated and precise. In this study a previously developed crop estimation model [23] is used to provide the number of OSR flowering pixels generated by this NDYI iso-cluster approach and we demonstrate its potential to estimate the OSR seed yield at the early flowering stage for the first time.

The aims are therefore (a) to evaluate the performance of a UAV with a MSI camera to estimate OSR canopy height across the different phenological growth stages over an 11 month growth cycle (b) to predict the final OSR seed yield (in mid-August) using NDYI, with a simplified pixel based iso-cluster classification method, during the flowering stage (May), and (c) to assess the accuracy of the predicted yield.

## Experimental

### Airy holme farm

Winter oil seed rape (codex) was planted at a rate of 76 seeds / m^2^ using a Claydon Hybrid T4 trailed drill (Rickerby, Hexham, UK), pulled by a Claas ARES 836 RZ tractor (Rickerby, Hexham, UK). The seeds were planted to a depth of 10 mm on the 1 August 2020, in an 8.1-hectare field, known locally as Waskerley Edge. The field was treated as follows, in general terms, for protection of the oilseed rape. At the sowing stage in August, a specific herbicide for oilseed rape was applied, to control invasive broadleaved and grass weeds (Banastar®,BASF Agricultural Solutions UK, Cheadle, Cheshire, UK) along with an adjuvant (Grounded AD®,Helena Agri-Enterprises, LLC, Collierville, USA). Additionally, and within days, slug pellets were applied to the field to protect the recently deployed sown seed. In September, an oilseed rape insecticide (Kung Fu®, Syngenta UK Ltd., Fulbourn, UK) was applied, to prevent damage from the cabbage stem flea beetle, along with an adjuvant (Activator® 90, Nutrien Ag Solutions, Loveland, USA). Subsequently, and additionally, a post emergence herbicide was applied during the winter (Falcon®, Adama Agricultural Solutions UK Ltd., Reading, UK) and spring (Korvetto®, Corteva Agriscience, Cambridge, UK). To restrict winter growth, a growth regulator was applied in December (Caryx®, BASF Agricultural Solutions UK, Cheadle, Cheshire, UK). In early spring, March, a wide-ranging fungicide (Protefin, Clayton Plant Protection, Dublin, Ireland) was applied, followed in late spring, May, with a further fungicide (Recital®, Bayer Crop Science Ltd., Cambridge, UK) along with an adjuvant (Roller, Agrovista UK Ltd., Nottingham, UK). Finally, 21 days prior to harvesting of the oil seed rape a pre-harvest desiccate was applied (Roundup Vista plus, Bayer Crop Science, Cambridge, UK).

To promote growth of the oil seed rape, fertilizer in the form 20% nitrogen, 8% phosphorus, 12% potassium and 7% sulfur trioxide (CF Fertilisers UK Ltd., Billingham, UK) was applied twice on the field spring 2021 (March and April). Additional nutrients were also added in spring (April–May) and included supplements for boron and molybdenum (Lebosol® MoBo, Lebosol® Dunger GmbH, Elmstein, Germany), phosphate, potassium and magnesium (Yaravita Magphos K, Yara Ireland, Grimsby, UK) and plant based amino acids and trace elements (Terra-Sorb Foliar, Agrovista UK Ltd.).

### Waskerley edge agricultural field

Soil analysis was done on this agricultural field in March 2021 (by Lancrop Laboratories, Pocklington, UK in association with Agrovista UK Ltd., Nottingham, UK). Soil analysis indicated that the sandy silt loam (with a composition of 49.1% sand, 42.3% silt and 8.6% clay) with a pH of 7.1, organic matter (5.4%) and a cation exchange capacity of 12.3 meq/100 g had normal soil levels of the major nutrients i.e. phosphorus (30 ppm), potassium (185 ppm) and magnesium (69 ppm) alongside secondary and micronutrients i.e. calcium (2120 ppm), copper (3.3 ppm), iron (735 ppm) and zinc (5.2 ppm). However, deficiencies were noted in term of secondary and micronutrients, specifically, sulfur (3 ppm) against a guideline of 15 ppm; sodium (14 ppm) against a guideline of 90 ppm; boron (1.3 ppm) against a guideline of 2.1 ppm; manganese (58 ppm) against a guideline of 75 ppm; and, molybdenum (0.01 ppm) against a guideline of 0.3 ppm. Therefore, supplements were added to the field to rectify these deficiencies. Further soil analysis, provided data that indicated a total nitrogen content of 0.258% and a carbon: nitrogen ratio of 12:2.

### Crop harvest

The crop was harvested on the 15 August 2021 using a Claas Lexion 570, Terra-Trac combine harvester with header attachment (Rickerby, Hexham, UK). Sensors (FarmTRX, Troo Corp., Ottawa, Canada), mounted on the upside of the grain elevator, to measure the amount of seeds recovered, by weight. The weight of seed is recorded on a data logger which transmits location and yield using GPS technology using on-board data processing software.

### Unmanned aerial vehicle

A multirotor UAV (DJI Phantom 4, supplied by Coptrz Ltd., Leeds, UK) was used with a multispectral camera with a 5 camera-array, stabilized with a 3-axis gimbal, covering the blue (450 ± 16 nm), green (560 ± 16 nm), red (650 ± 16 nm), red edge (730 ± 16 nm) and near-infrared (840 ± 26 nm) spectra with an additional camera that provides images in RGB (visible) mode [23]. The camera was angled perpendicular to the ground, with data capture occurring in hover and capture mode. Images were captured as 16-bit TIF files corrected for ambient radiance values. The UAV speed was 5.0 m/s at an average flight height of 50.6 m, and a resolution of 2.7 cm/px. All other settings were as manufacturer’s recommendations. Specific weather conditions relating to daytime temperature during flight, wind speed and direction were recorded using a handheld anemometer (Benetech^®^ GM816, Amazon UK)), and supplemented with UAV pilot anecdotal observations on cloud coverage and logged per flight. In total, all data reported was based on 10 UAV flights between September 2020 and August 2021.

### UAV photogrammetric processing

**Fig 2:**
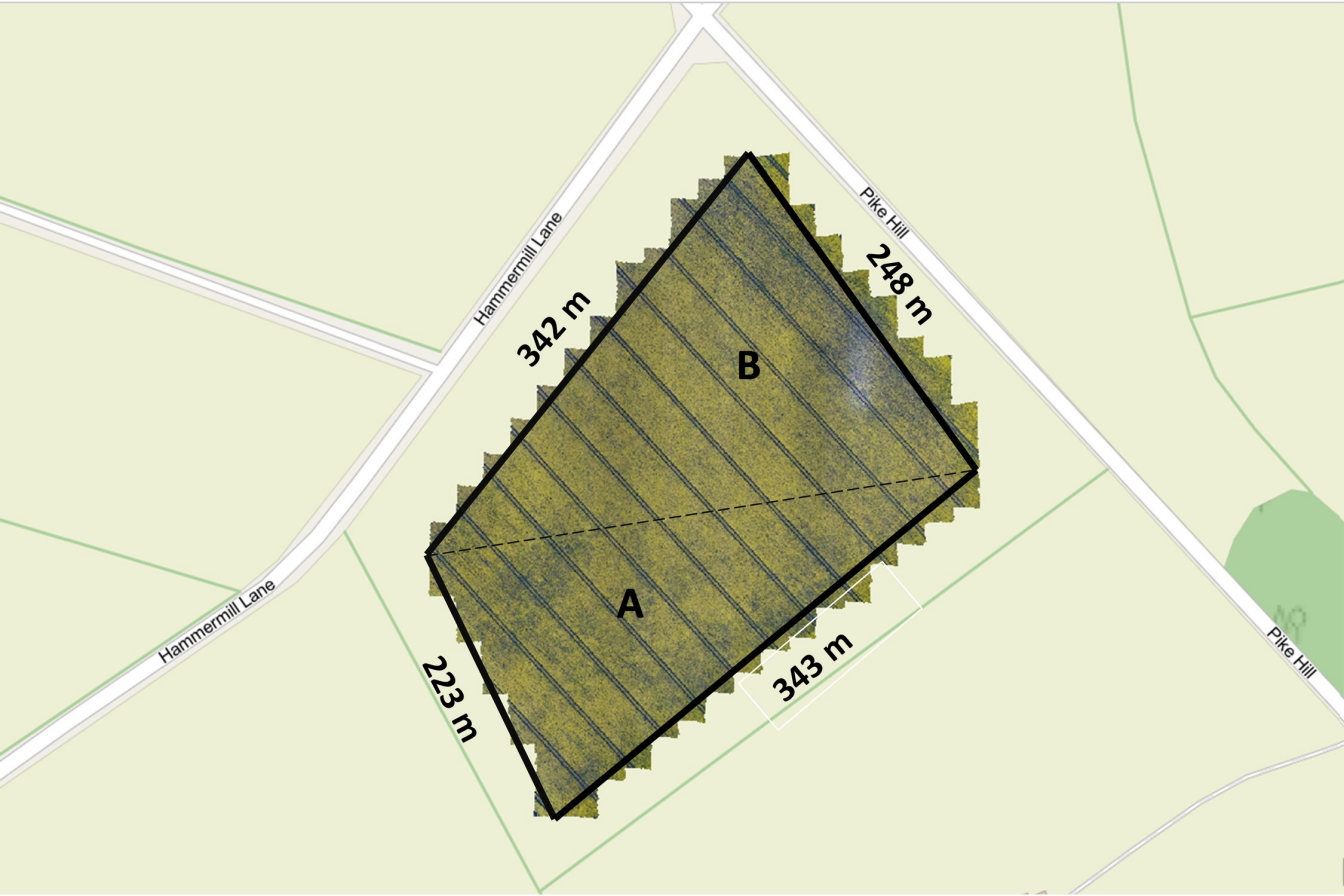
Orthomosaic image of oilseed rape field. [Note: The chosen area to investigate is outlined with an approximate area of 8.1 ha (80,653 m^2^). Area of A = 3.82 ha |(38,245 m^2^) and Area of B = 4.24 ha (42,408 m^2^)].

The multispectral UAV images were used to build an orthomosaic image using Agisoft Metashape Professional (64 bit) software v.1.7.1 (Agisoft LLC, St. Petersburg, Russia). The UAV photogrammetric processing steps were as follows: the aerial images were merged and aligned to construct a sparse point cloud by matching similar image attributes. The images were then accurately positioned to generate a 3D point cloud based on the GPS coordinates of each image and, a solid mesh model was created. Finally, an orthomosaic image, shown in Fig 2, was built using the WGS 1984 Web Mercator coordinate system.

### Plant height estimation

**Fig 3:**
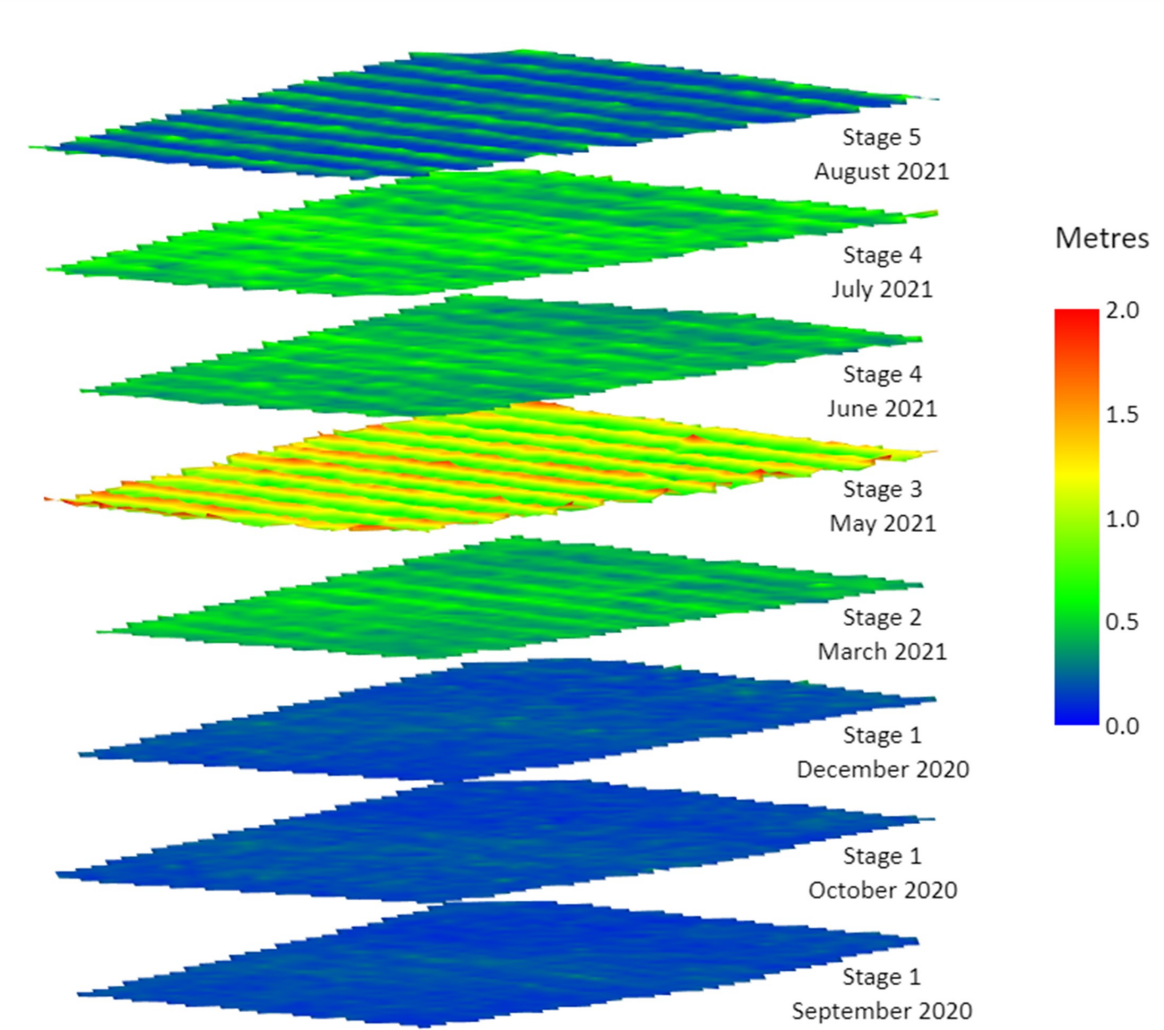
3-Dimensional canopy height model of oilseed rape at the five phenological growth stages.

**Fig 4:**
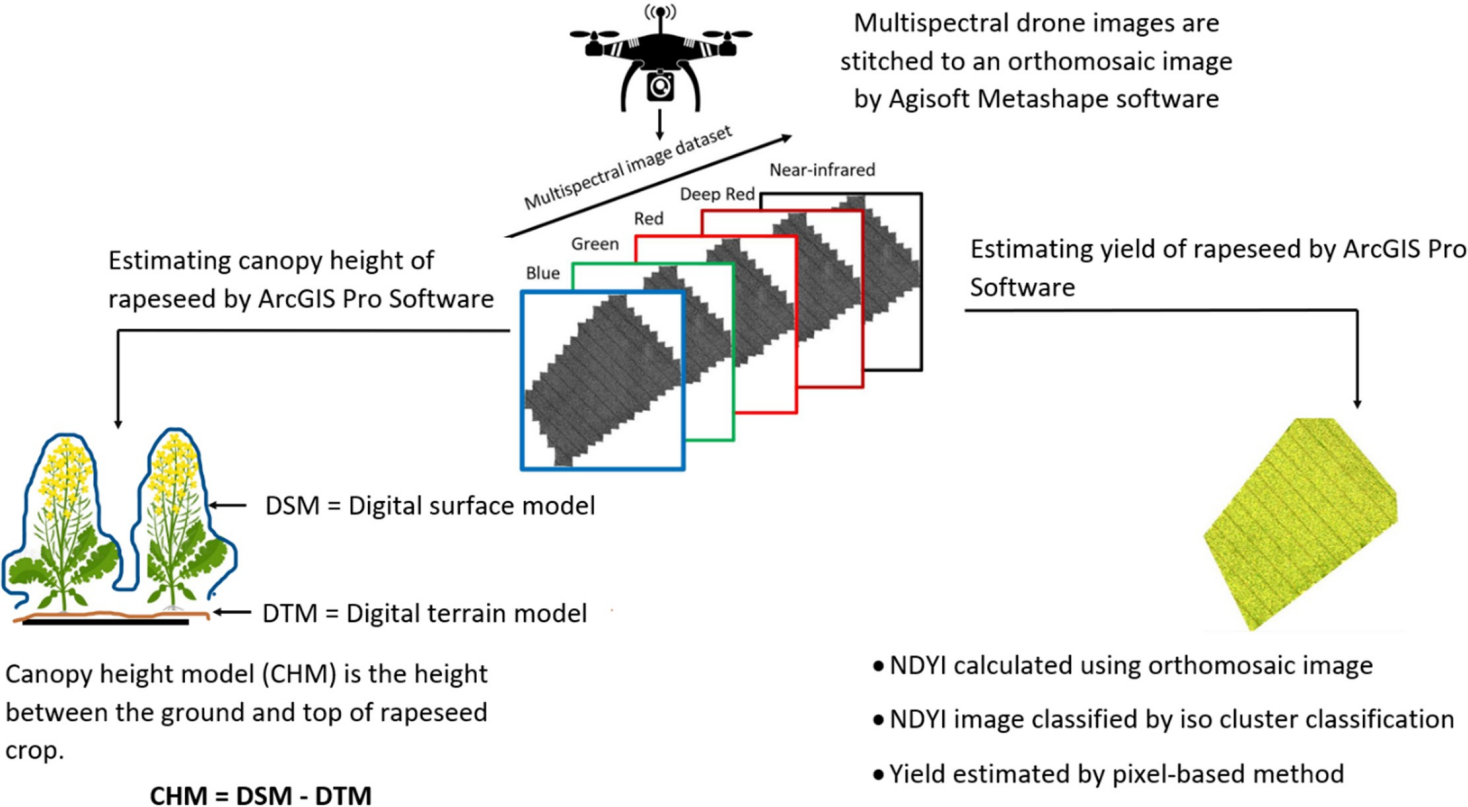
Workflow of data processing to derive the canopy height model (CHM) and normalised difference yellow index (NDYI) maps.

The OSR height estimation was performed on ArcGIS Pro v.2.8.0 software (Esri Inc., West Redlands, CA, USA). ArcGIS Pro software can construct a canopy height model (CHM) based on the principle of structure from motion (SfM). The SfM photogrammetry creates a rigid 3D model by matching similar features of several overlapping images [8]. The 3D point clouds, built by Agisoft, were extracted and used by ArcGIS Pro to create CHMs based on the time-series data, relating to the rapeseed phenological growth stages, Fig 3. [Note: only 8 flights are represented in Fig 3; in May and June two flights were done, per month, but with no significant differences noted in terms of growth within the short timeframe represented.] The 3D point clouds were used to generate a digital surface model (DSM) and a digital terrain model (DTM). The DSM is calculated by including features which are elevated from above the ground and the DTM is calculated by interpolation of features from either the ground or soil surfaces, as shown in Fig 4. The DSM and DTM generated for each spectral band were merged to produce an overall multispectral raster image. The CHM, which is the height between the ground and the top of the OSR crop, was then calculated using the raster calculator tool in ArcGIS (CHM = DSM ‐ DTM).

### Vegetation index calculation and Iso-cluster classification

ArcGIS Pro software was used for vegetation index calculation and iso-cluster classification, Fig 4. The normalised difference yellow index (NDYI) raster was derived by calculation of the reflectance of green and blue orthomosaic image using the raster calculator tool.


$$\text{NDYI=}\;\frac{\text{(Green-Blue)}}{\text{(Green+Blue)}}$$ (1)

The NDYI captures the yellowness of the OSR flowers utilising the green and blue spectral bands. Therefore, the NDYI was calculated on two image datasets of OSR flowering in May i.e. 14 and 27 May 2021. The NDYI raster was further classified by iso-cluster classification. Iso-cluster classification is an unsupervised classification tool in ArcGIS pro which automatically groups similar clusters to output a classified image. It uses a similar principal to k-means clustering where centroids are placed according to the number of clusters assigned. The Euclidian distance for each pixel with the respective centroid is calculated. The pixels for each cluster are classified on its closest Euclidian distance into separate clusters which each cluster having a similar value. Five clusters were assigned to classify the NDYI raster. The first cluster had pixels representing the soils surface, while the second cluster include pixels representing green vegetation. The remaining three clusters represented rapeseed flower pixels of varying yellowness.

### Oilseed rape seed yield estimation

The basis of the OSR seed yield estimation, is based on the principle that the OSR flowers will ultimately convert into ripened pods, after continued fertilizer and soil nutrients interventions by the farmer. Therefore, it can be approximated that the number of pixels representing the OSR flowers, by NDYI iso-cluster classification are an estimate of the OSR seed yield. By using a simplified pixel-based approach, as originally proposed by [24], an estimate of OSR seed yield, in units of t/ha, can be made using a weighting factor, F, which is the ratio of the OSR flower pixels to the total number of pixels for the field. And secondly, using a heuristic approach to estimate crop yield, as practiced by farmers, that approximates the area of the OSR flowers to a mass of OSR seeds, P(OSR), and that has been developed and applied by other researchers [24]:

$$\text{Estimated}\;\text{seed}\;\text{yield}\;\text{(t/ha)}\;=\;\frac{F\;x\;P(OSR)}{A}$$ (2)

Where, F = rapeseed flower pixels / ∑ pixels; P (OSR) = area of OSR flowers in the field, calculated by multiplying the number of pixels of OSR flowers by the resolution of the drone images i.e. (0.027 m)^2^ / pixel, and approximating that to a mass (t); and A = Area of the field in ha.

**Fig 5:**
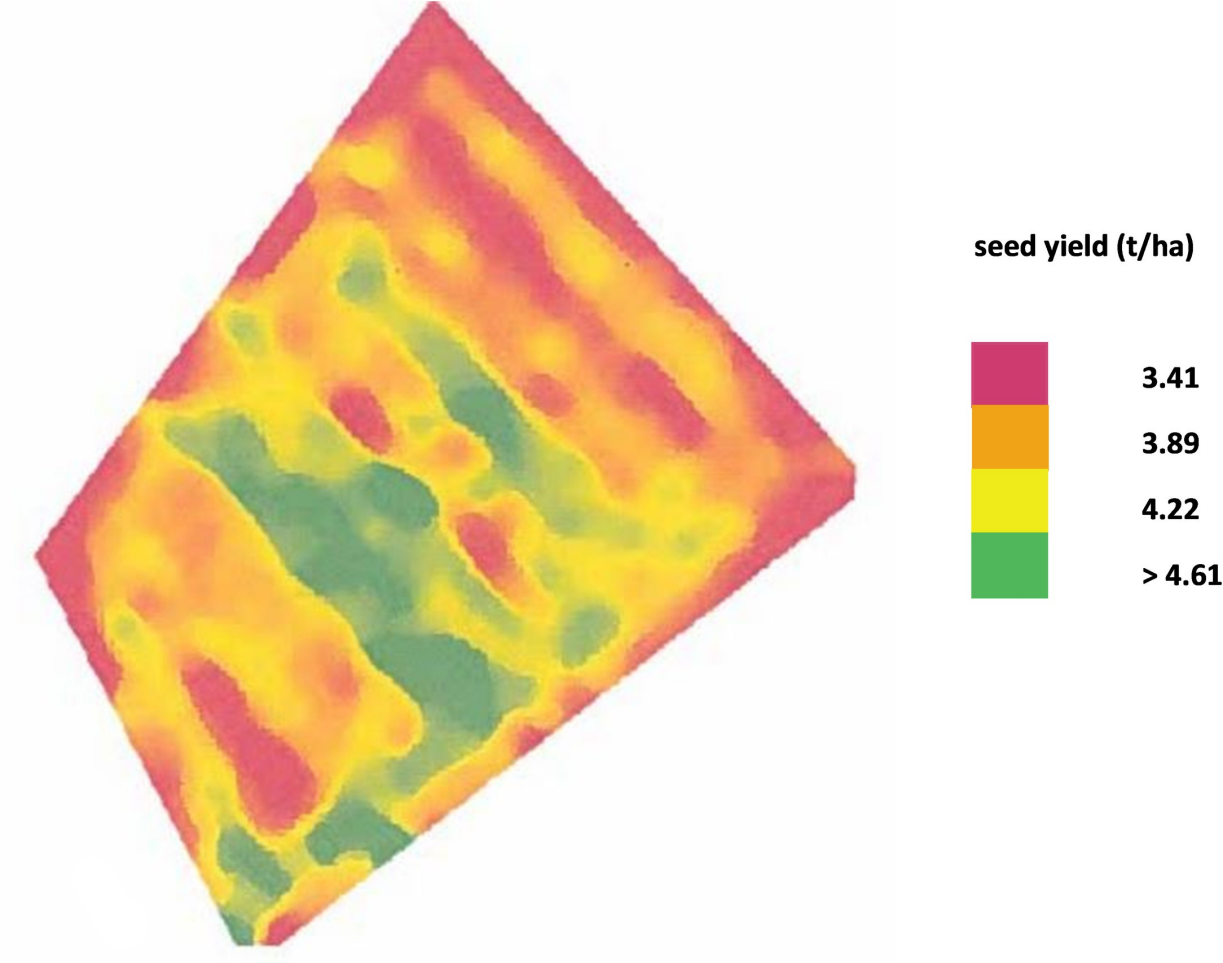
Actual seed yield map of oilseed rape. [Note: the reported field area is 8.15 ha (81,500 m^2^)].

**Fig 6:**
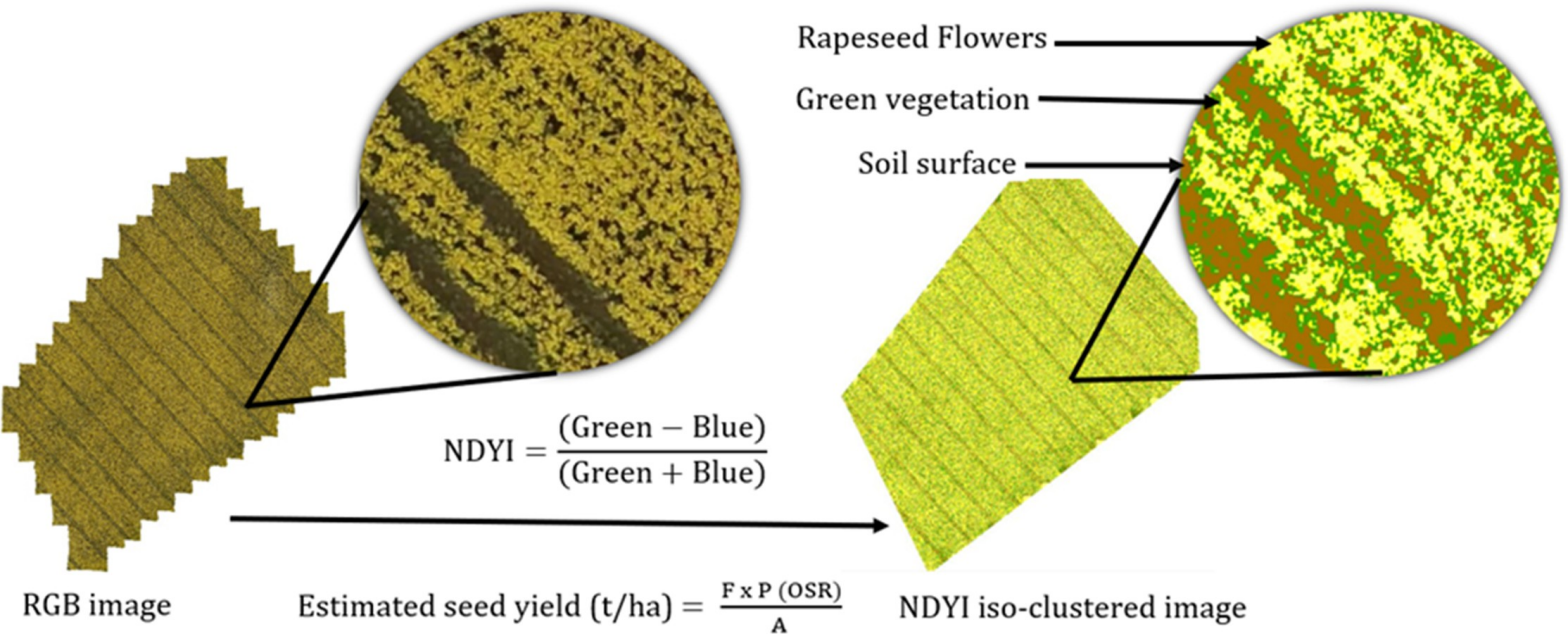
Normalised difference yellow index (NDYI) iso-clustered image representing the oilseed rape flowers, green vegetation, and soil surface.

According to the actual corrected seed yield map, shown in Fig 5, supplied by FarmTRX (Troo Corp., Ottawa, Canada), some variation of yield is noted across the 8.15 ha. The yield map has variation across its area, highlighted as follows: low yield (red = 3.41t/ha), medium (orange = 3.89 t/ha), high (yellow = 4.22 t/ha) and very high (green > 4.61 t/ha) yield areas, and shown in Fig 5. As a result, and with reference to the corrected yield map (FarmTRX), the predicted seed yield based on the NDYI iso-cluster image was determined in 25 distinct places for the four identified yield sites i.e. low, medium, high and very high yield, Fig 6.

### Statistical analysis

To analyse the link between the estimated seed yield and the actual yield, Pearson correlation and root mean square error (RMSE) calculations were made. The larger R^2^ and low RMSE will indicate the higher precision and accuracy of the estimated yield model.

$$\text{RMSE}\;=\;\sqrt{\frac{\sum_{i=1}^{n}(yi-\hat{yi})}{n}}$$ (3)

where *yi* = estimated yield; $\hat{yi}$ = actual yield; and *n* = number of observations.

## Results and discussion

### Estimation of OSR plant height by multispectral-UAV at the various phenological growth stages

Multispectral-UAV estimates of plant height from the CHM were conducted on an 8.1 ha of OSR field, Fig 2. This study has no ground-truth data of OSR crop height, over the five stages, to validate the UAV data. Therefore, the UAV images were validated by measuring the height of the wall adjacent to the OSR field. Statistical analysis (t-test) was performed on manually measured wall heights (1.13 ± 0.13 m, n = 13) against the UAV-MSI estimated wall height (1.18 ± 0.22, n = 13); both height determinations were done using 13 repeats, of different parts of the wall, on the same date. It was found that no statistical difference was noted between the two measurements (at a p-value of 0.49, 95% confidence interval), and the estimated OSR plant height measurements, as estimated from UAV-MSI images are used as accurate for the rest of the study.

**Fig 7:**
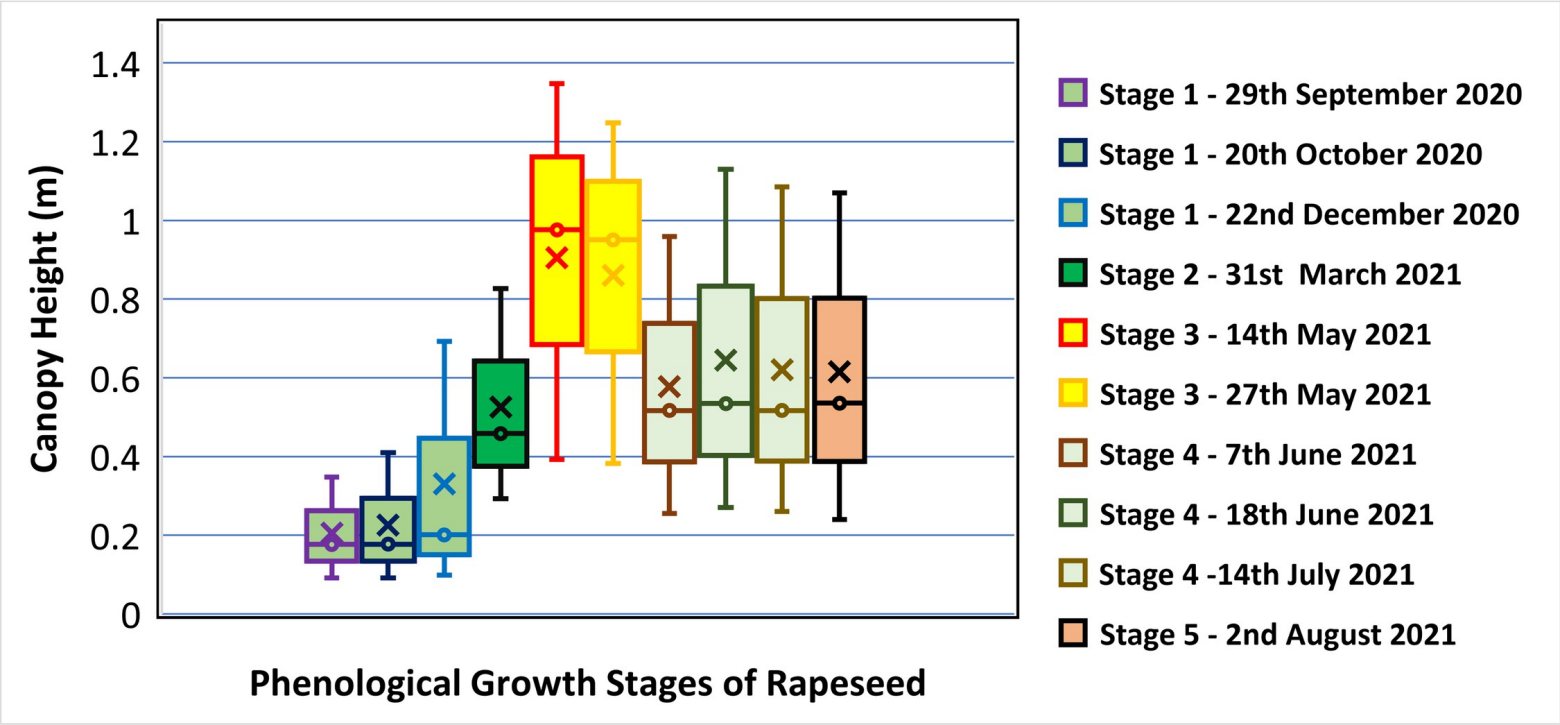
Determined oilseed rape canopy height at the five phenological growth stages. [Note: the limits of the box represent the upper and lower quartile of the data assessed at the 95% confidence limit while the whiskers show the minimum and maximum heights determined. The horizontal line within the box represents the median height while the cross is the mean height].

**Fig 8:**
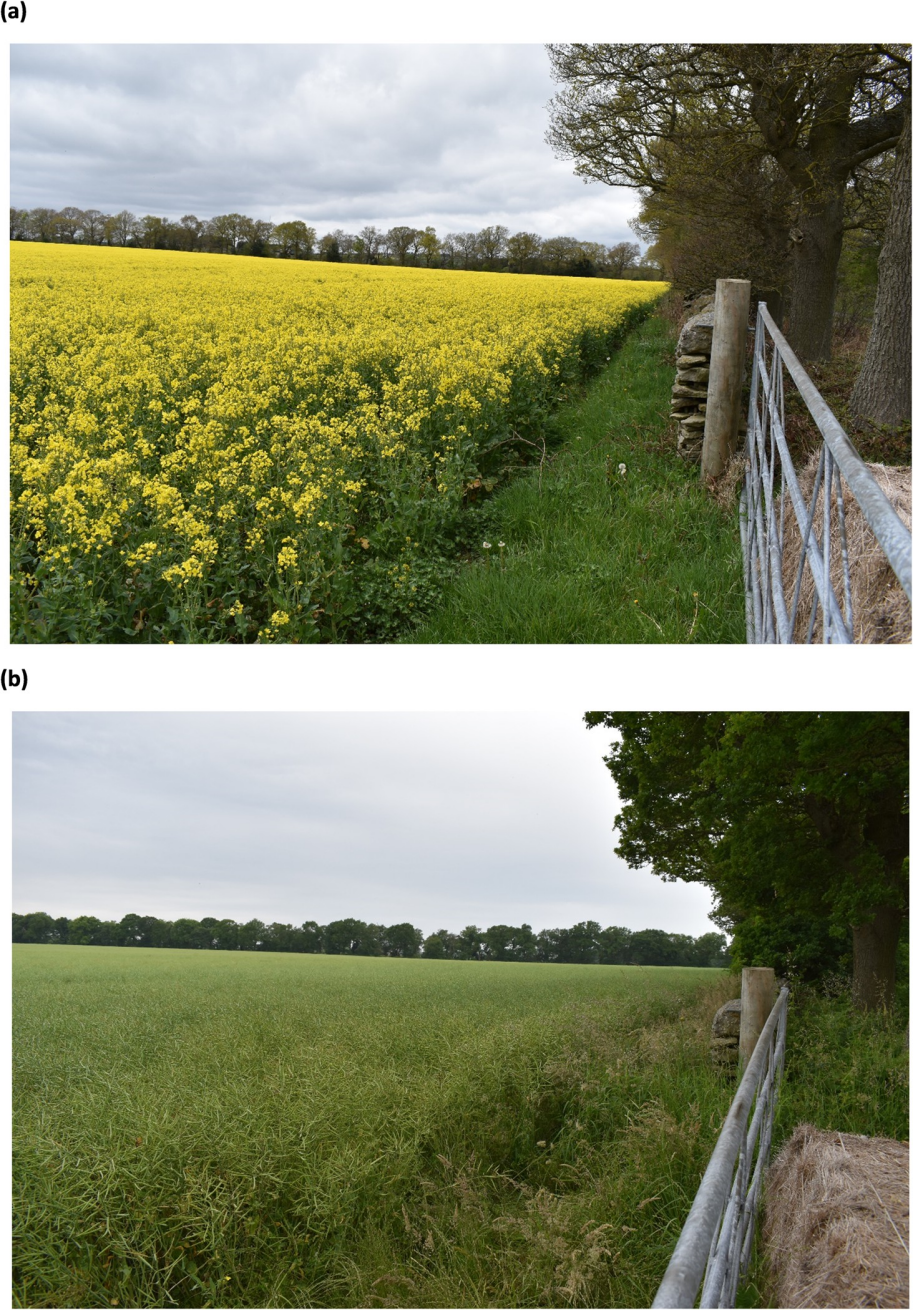
Photographs of the oilseed rape crop on the (a) 14 May 2021 during stage 3 (flowering), and (b) 14 July 2021 during stage 4 (podding).

A boxplot statistically representing the distribution of numerical data with minimum, median, mean and maximum OSR plant height across the field is shown in Fig 7. In stage 1 (Rosette), the OSR height has values of 0.092 m, 0.21 m and 0.35 m (minimum, mean and maximum). In stage 2 (Bolting), the OSR height has values of 0.29 m, 0.53 m and 0.83 m (minimum, mean and maximum). In stage 3 (Flowering), the OSR height has values of 0.39 m, 0.91 m and 1.35 m (minimum, mean and maximum). In stage 4 (Podding), the OSR height has values of 0.26 m, 0.58 m and 0.96 m (minimum, mean and maximum). In stage 5 (Ripening), the OSR height has values of 0.24 m, 0.62 m and 1.07 m (minimum, mean and maximum). It can clearly be seen, Fig 7, that the highest OSR plant height was achieved at stage 3, the flowering stage. Further, the decline of OSR height after flowering (stage 3) can be due to the challenging canopy architecture of the OSR plant. At the flowering stage, the canopy height is measured precisely due to the inflorescence which can be visible on aerial images. However, after the flowering stage, when the OSR starts podding, the pods become heavier, and the plants slump whilst accommodating the extra weight. This is illustrated using the photographs in Fig 8, that show the full height of the crop at stage 3 (flowering), Fig 8A compared to the pod-laden OSR crop at stage 4, podding, Fig 8B. Therefore, it becomes challenging to estimate the plant dimensions (rather than their height above ground level) accurately using the CHM generated from UAV images.

As reported in other studies, and in agreement with our data, the main stem of the OSR plant reaches between 30–60% of its maximum length at stage 2 (Bolting) [3]. Additionally, the OSR plant achieves its full height in the flowering stage (stage 3), with an average plant height of between 0.75 m to 1.75 m. Similarly, a study used a UAV with RGB sensors to estimate winter OSR crop height which they found varied between 0.80 m and 1.6 m [7]. However, it should be noted that differences in OSR plant height data will vary, based on its geographical location along with variation in soil type and fertilizer and other treatment variations, as well as climatic conditions will all influence OSR growth and development. It is recommended therefore that localised geographical area phenotyping is done in relation to precision agriculture practices but are informed by pseudo-timeframe UAV-MSI data informatics.

### Correlation study between estimated and actual seed yields

**Fig 9:**
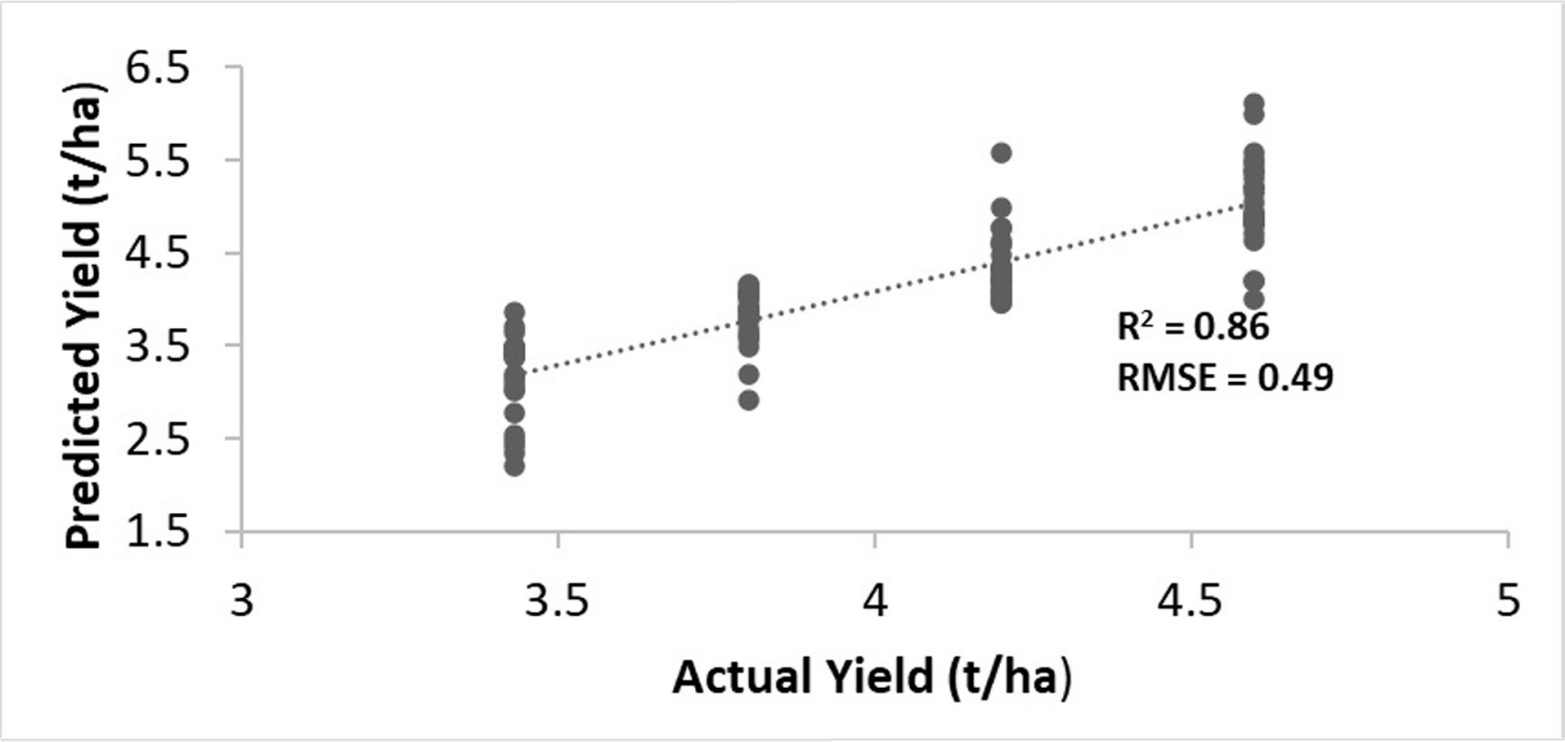
Correlation study between the estimated and actual seed yields.

Initially, two dates in May 2021 (14 and 27 May) were chosen by visual inspection of the OSR inflorescence in the RGB images to determine the estimated seed yield, Fig 9. The NDYI maps were classified into five clusters by iso-cluster classification. The classified clusters were grouped as soil surface, green vegetation, and OSR flowers, Fig 6. If the NDYI value of a cluster pixel was more than 0.12 reflectance it was classified as OSR flowers. In this manner, three clusters were identified to have NDYI pixel values greater than 0.12. All three clusters were grouped as rapeseed flowers. Similarly, the NDYI iso-clustered pixel values for green vegetation was in the range between -0.15 to -0.45 and soil surface was in the range between -0.45 to -1. Using a similar approach on winter OSR [25], this method identified flowers with NDYI pixel values greater than 0.28. In contrast, their NDYI values were significantly lower for green vegetation and soil surface regions, thus representing the need for localised monitoring and determination. The classified OSR flower pixels, by NDYI iso-cluster classification, was incorporated in Eq 2, to estimate the OSR seed yield. The results, shown in Fig 9, from our study demonstrates the strong correlation (R^2^ = 0.86 and RMSE = 0.49)) between the estimated seed yield and the actual OSR seed yield. It is noted however, that while this NDYI model assumes a 100% conversion of a flower pixel to yield that is unlikely to be correct. Indeed, previous studies [26] using ground truth data, that the conversion from flowers to pods is actually 75%.

## Conclusion

The results of this work provide an effective and simple approach for classification of OSR flowers as an estimate of seed yield. Our new approach, using an automated iso-cluster classification model, has proven to be an effective estimator of seed yield and with good accuracy and precision. Future work will seek to evidence the robustness of the iso-cluster classification approach for assessing crop yield based on the flowering stage, for both the same and other widely grown crops. In addition, the effective use of a calibrated UAV-MSI system can estimate crop height to not only follow, over an 11-month period, the development of OSR, but also assess the point of optimum flowering for estimation of seed yield 3 months ahead of harvesting. The deployment of this approach in precision agriculture, without the need for challenging computer programming, could be a convenient and effective approach to be used by agronomists, as well as provide farmers with insight into estimated OSR seed yield 3 months ahead of harvesting. This could be an invaluable asset to farmers as it would allow the sale of the OSR crop in advance of harvesting to solicit the best price.
